# Beyond infection—a ferritin-LDH-IL-10 triad to unmask intravascular lymphoma in fever of unknown origin: a case report and literature review

**DOI:** 10.3389/fmed.2026.1818936

**Published:** 2026-04-24

**Authors:** Yuanyuan Chen, Wentao Ni, Yuxin Wang, Yuan Li, Xun Wang, Dingbao Chen, Yan Gao

**Affiliations:** 1 Department of Infectious Diseases, Peking University Hepatology Institute, Peking University People’s Hospital, Beijing, China; 2 Department of Pulmonary and Critical Care Medicine, Peking University People’s Hospital, Beijing, China; 3 Department of Gastrointestinal Oncology, Key Laboratory of Carcinogenesis and Translational Research (Ministry of Education/Beijing), Peking University Cancer Hospital and Institute, Beijing, China; 4 Department of Nuclear Medicine, Peking University People’s Hospital, Beijing, China; 5 Department of Thoracic Surgery, Peking University People’s Hospital, Beijing, China; 6 Department of Pathology, Peking University People’s Hospital, Beijing, China

**Keywords:** case report, fever of unknown origin, IL-10, intravascular large B-cell lymphoma, PET-CT, targeted lung biopsy

## Abstract

**Objective:**

To highlight the diagnostic value of the ferritin-LDH-IL-10 triad and image-guided targeted biopsy in intravascular large B-cell lymphoma (IVLBCL) presenting as fever of unknown origin (FUO), with a review of relevant literature.

**Methods:**

We present a case of a 60-year-old woman with a 10-month history of cough, progressive fever, and weight loss. We retrospectively analyzed her clinical course, laboratory profile, imaging findings, and histopathological results, and reviewed the relevant literature on IVLBCL diagnosis.

**Results:**

Extensive initial investigations including multiple biopsies (bone marrow, colon, skin) and imaging were nondiagnostic. Persistent extreme hyperferritinemia (peak >15,000 ng/mL), markedly elevated LDH (735 U/L), and strikingly high IL-10 (>600 pg./mL) formed a diagnostic triad raising suspicion for IVLBCL. Re-review of PET-CT identified a subtle pulmonary FDG-avid focus (SUVmax 3.0), guiding video-assisted thoracoscopic wedge resection. Histopathology confirmed IVLBCL with CD20 + tumor cells within vascular lumina. The patient subsequently met criteria for secondary hemophagocytic lymphohistiocytosis. R-CHOP immunochemotherapy achieved rapid symptom resolution and complete metabolic response on follow-up PET-CT.

**Conclusion:**

The triad of extreme hyperferritinemia, elevated LDH, and markedly increased IL-10 may serve as a useful diagnostic clue to raise suspicion for IVLBCL in FUO patients, though its interpretation is confounded by secondary HLH and requires prospective validation. When random biopsies fail, multidisciplinary re-evaluation of PET-CT to identify subtle targets for image-guided biopsy is essential for definitive diagnosis. Early recognition of this triad and adoption of a hypothesis-driven biopsy strategy can significantly improve diagnostic outcomes in this elusive lymphoma.

## Introduction

Intravascular large B-cell lymphoma (IVLBCL) is a rare and aggressive extranodal lymphoma characterized by the selective growth of malignant B-cells within the lumina of small to medium-sized blood vessels, sparing larger vessels and lymph nodes (1, 2). This unique growth pattern results in an absence of mass lesions or lymphadenopathy, making diagnosis exceptionally challenging. IVLBCL classically presents with fever of unknown origin (FUO), nonspecific systemic symptoms, and multiorgan involvement, often mimicking infections or inflammatory disorders (3). The diagnosis is frequently delayed or missed, leading to poor outcomes. We report a case of IVLBCL presenting as FUO, where a distinctive triad of laboratory abnormalities—extreme hyperferritinemia, elevated lactate dehydrogenase (LDH), and markedly increased interleukin-10 (IL-10)—guided the diagnostic workup. This case highlights the critical role of this biomarker triad and multidisciplinary, image-guided biopsy in achieving a timely diagnosis. We also review the literature on diagnostic strategies for IVLBCL, with emphasis on biomarker interpretation and imaging-guided biopsy approaches.

## Case presentation

A 60-year-old woman was transferred to our Infectious Diseases Department in July 2025 with a 10-month history of cough and progressive fever. Her illness had begun in September 2024 with a non-productive cough, which by March 2025 was accompanied by daily afternoon fevers (up to 38.7 °C), chills, and unintentional weight loss of 10 kg.

Her past medical history included cervical carcinoma *in situ* treated with conization 20 years ago, thyroid adenoma treated with left thyroidectomy 17 years ago (on levothyroxine 25 μg/day), and hyperlipidemia managed with lovastatin. Family history was unremarkable. She had traveled to Thailand and Hainan in 2024 but reported no known infectious exposures.

**Figure 1:**
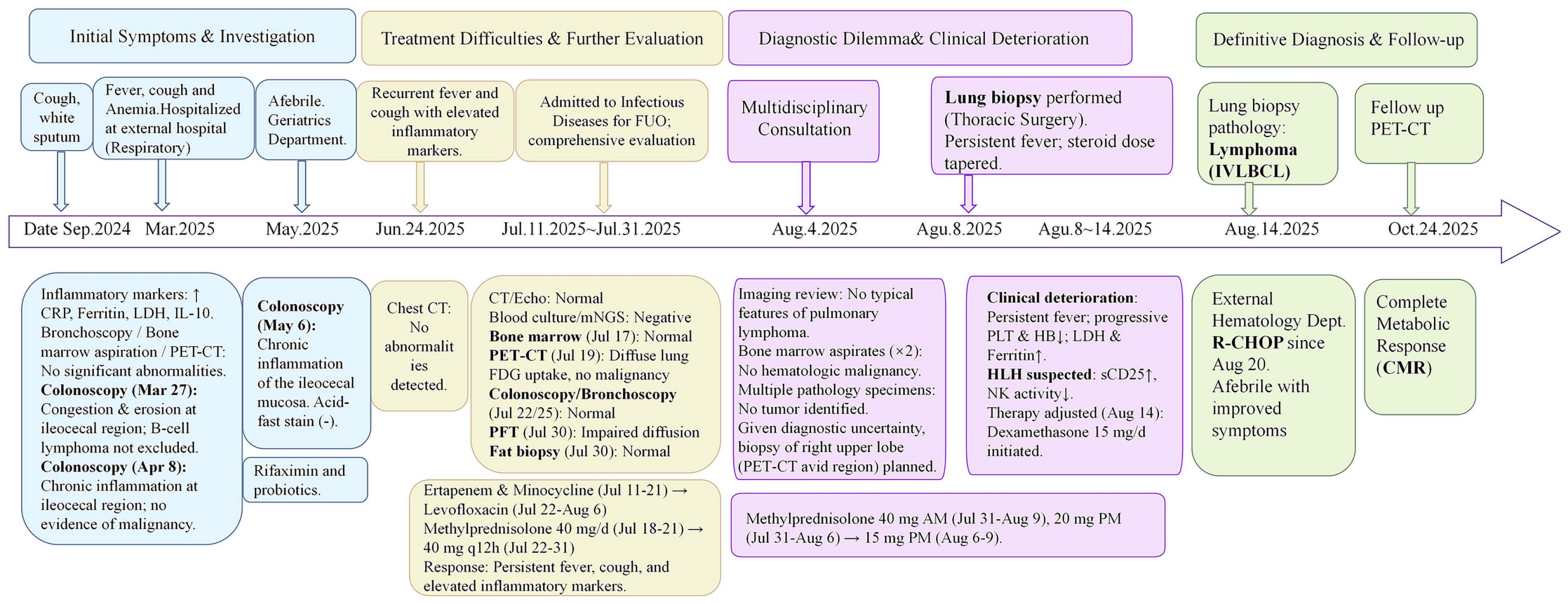
Diagnostic timeline and management of intravascular large B-cell lymphoma (IVLBCL) presenting as fever of unknown origin. This timeline illustrates the patient’s 10-month diagnostic odyssey, divided into four phases: Phase 1: Initial presentation and extensive evaluation (Sep 2024–Jul 2024). The patient presented with persistent cough, fever, and anemia. Serial investigations—including bronchoscopy, colonoscopy, bone marrow biopsy, and whole-body PET-CT—were nondiagnostic. Phase 2: Escalating inflammation and diagnostic impasse (Jul 2025–early Aug 2025). A severe systemic inflammatory state emerged, marked by extreme hyperferritinemia, elevated LDH and IL-10, and impaired diffusing capacity despite normal chest CT. Multiple random biopsies remained negative. Secondary hemophagocytic lymphohistiocytosis (HLH) criteria were met. Phase 3: Targeted re-evaluation and biopsy (Aug 2025). Driven by high suspicion for IVLBCL, prior PET-CT was re-reviewed, revealing a subtle FDG-avid pulmonary focus (SUVmax 3.0). This guided a video-assisted thoracoscopic wedge resection. Phase 4: Diagnosis and treatment response (Aug 2025–Oct 2025). Lung biopsy confirmed IVLBCL with intravascular lymphoid cells. R-CHOP immunochemotherapy led to rapid symptom resolution. Follow-up PET-CT showed complete metabolic response (CMR). Abbreviations: CMR, complete metabolic response; FDG, fluorodeoxyglucose; HLH, hemophagocytic lymphohistiocytosis; IL-10, interleukin-10; IVLBCL, intravascular large B-cell lymphoma; LDH, lactate dehydrogenase; PET-CT, positron emission tomography–computed tomography; SUV, standardized uptake value.

Initial evaluation at an external hospital between March and April 2025 revealed anemia (hemoglobin 83 g/L) and marked inflammation: ferritin 3,448 ng/mL, LDH 585 U/L, IL-6 50.9 pg./mL, and IL-10772 pg./mL. Extensive infectious workup was negative. Bronchoscopy, bone marrow aspiration, and whole-body PET-CT showed no definitive evidence of infection or malignancy. Three colonoscopies showed only chronic inflammation without malignancy. The patient was discharged after symptomatic improvement (Figure 1). The diagnostic timeline is summarized in Figure 1.

In late June 2025, her fever recurred, now persistent throughout the day and reaching 40 °C, accompanied by worsening cough, fatigue, anorexia, and transient left posterior cervical lymphadenopathy. She was readmitted to our service on July 11.

**Figure 2:**
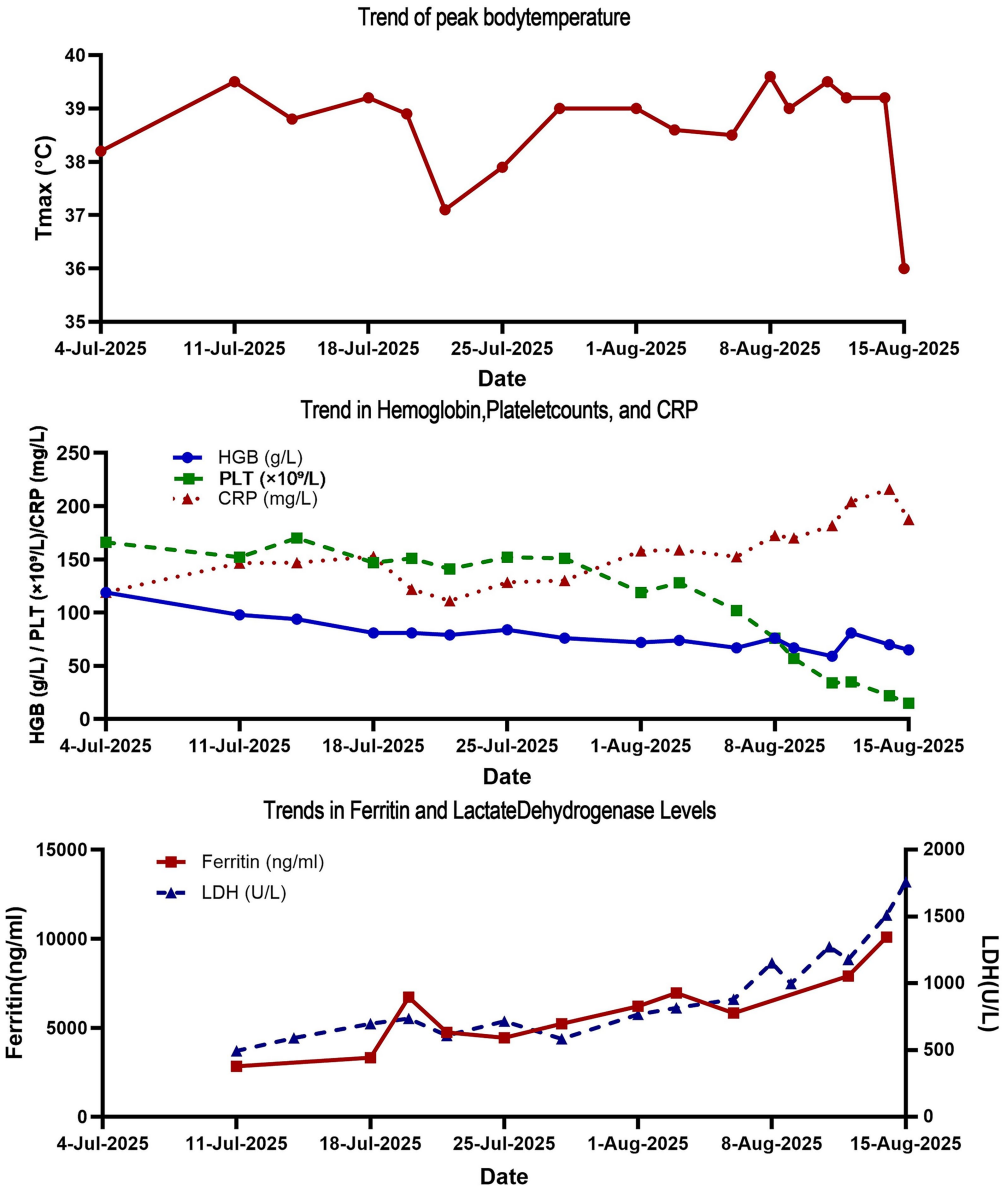
Dynamic laboratory profile during the diagnostic evaluation. The panel shows trends in peak body temperature (°C) over time; concurrent trends in key hematologic and acute-phase markers including hemoglobin (HGB, g/L), platelet count (PLT, ×10^9^/L), and C-reactive protein (CRP, mg/L); and trends in critical biomarkers of systemic inflammation and tissue damage including ferritin (ng/mL) and lactate dehydrogenase (LDH, U/L). The data illustrate the evolution of a profound inflammatory state and cytokine storm, characterized by progressive cytopenias, extreme hyperferritinemia, and elevated LDH, which were pivotal clues to the underlying lymphoproliferative disorder.

On admission, she appeared cachectic. Physical examination revealed crackles in the right lower lung field; there were no skin lesions or palpable lymphadenopathy. Laboratory studies showed progressive normocytic anemia (hemoglobin 94 → 76 g/L), thrombocytopenia (platelets 119 → 76 × 10^9^/L), and rising inflammatory markers: CRP 119 mg/L, ferritin 3,317 → 6,712 ng/mL, LDH 591 → 735 U/L, and IL-10664 pg./mL (Figure 2). Repeated infectious workups—including blood cultures, NGS of blood and bronchoalveolar lavage fluid (BALF), fungal markers, and tuberculosis testing—remained negative. Autoimmune and tumor marker panels were again unremarkable.

**Figure 3:**
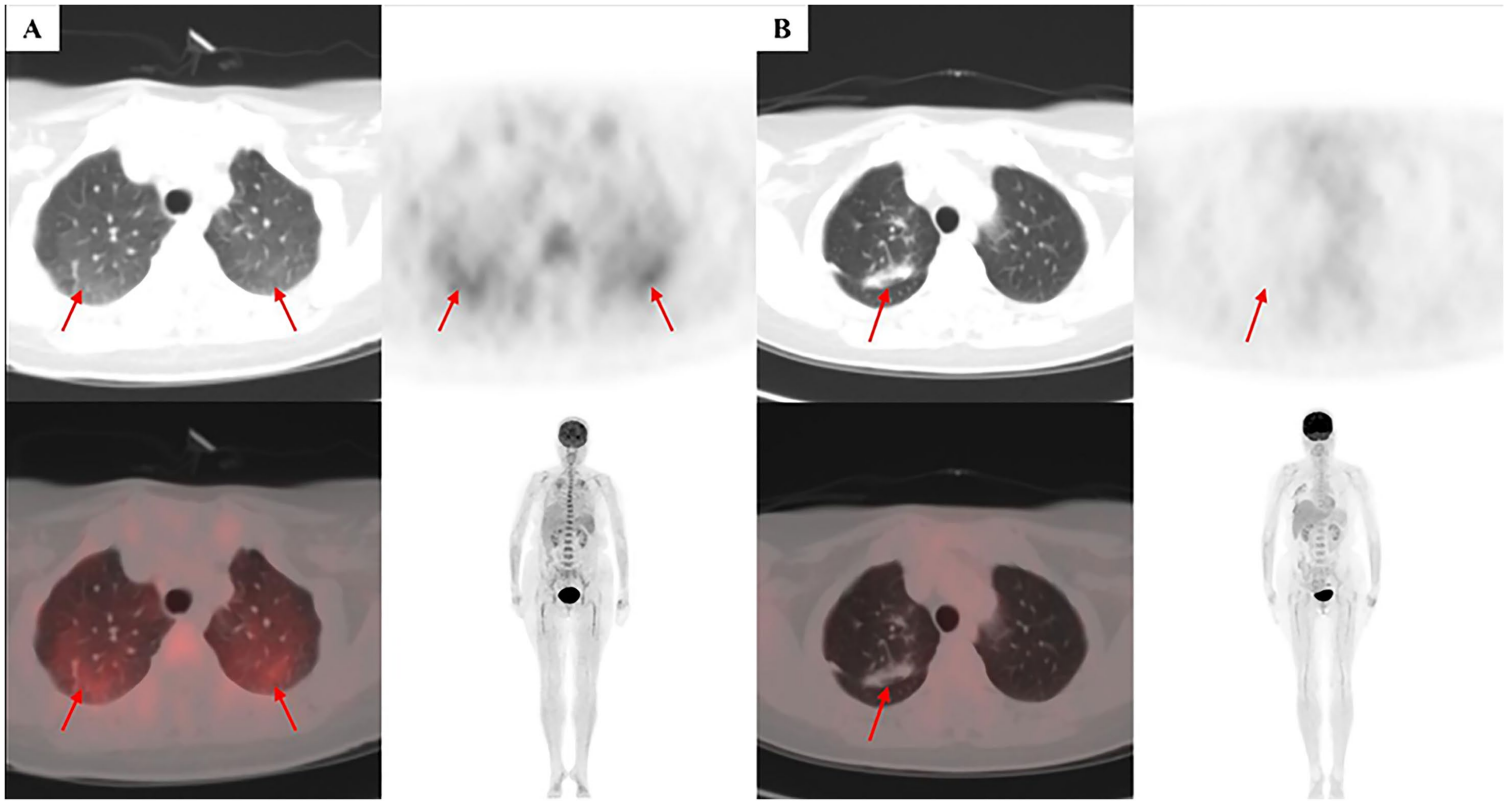
Positron emission tomography-computed tomography (PET-CT) findings at presentation and after treatment. **(A)** Initial scan (July 19, 2025) reveals diffuse, bilateral pulmonary fluorodeoxyglucose (FDG) uptake, most pronounced in the upper lobes (arrows), with a maximum standardized uptake value (SUVmax) of 3.0 in the upper lung fields versus 1.0 in the remaining parenchyma. **(B)** Follow-up scan (October 14, 2025) after systemic immunochemotherapy demonstrates complete resolution of the previously observed hypermetabolic foci, consistent with a complete metabolic response (CMR). The linear opacity in the right upper lobe (arrowhead) is compatible with post-surgical change following the diagnostic wedge resection.

Chest CT showed no parenchymal abnormalities. A PET-CT on July 19 demonstrated diffuse bilateral pulmonary FDG uptake, most pronounced in the upper lobes (right upper lobe SUVmax 3.0), with increased splenic and bone marrow activity but no discrete mass lesions (Figure 3A). Bronchoscopy on July 25 was negative, as was a repeat bone marrow aspiration on July 17 (morphology and flow cytometry normal; IgH gene rearrangement weakly positive in one tube only). A fourth colonoscopy on July 22 showed normal mucosa with chronic inflammation on biopsy. Pulmonary function tests on July 30 revealed mildly reduced diffusing capacity (DLCO 68% predicted) with normal spirometry. Random skin and fat biopsy on July 30 identified no tumor cells.

Empirical antibiotics (ertapenem, levofloxacin) produced no response. High-dose methylprednisolone (40 mg/day, then 40 mg q12h) from July 18 to 31 failed to control fever or improve inflammatory markers (Figure 1).

Throughout this process, a broad differential diagnosis was systematically considered. Infectious etiologies and autoimmune disorders were rigorously excluded by negative cultures, NGS, autoantibody panels, and lack of response to antibiotics and corticosteroids. Malignancy, particularly lymphoma, remained a leading consideration given the constellation of fever, weight loss, elevated LDH, and hyperferritinemia. However, extensive random biopsies (bone marrow, colon, skin) and two PET-CT scans failed to identify a tumor, leaving the diagnosis elusive.

On August 4, a multidisciplinary team (MDT) meeting was convened, bringing together specialists from Infectious Diseases, Nuclear Medicine, Respiratory Medicine, Thoracic Surgery, Hematology, and Pathology. The team re-reviewed all imaging and, noting the subtle but persistent FDG-avid focus in the right upper lobe (SUVmax 3.0), concluded that a targeted biopsy of this area was essential.

On August 8, the patient underwent video-assisted thoracoscopic wedge resection of the right upper lobe. Postoperatively, her condition deteriorated: she developed worsening cytopenias (hemoglobin 65 g/L, platelets 45 × 10^9^/L), ferritin rose to >15,000 ng/mL, and she met criteria for hemophagocytic lymphohistiocytosis (HLH) with fever >38.5 °C for >7 days, cytopenias, hyperferritinemia, elevated sCD25 (44,891 pg./mL), and decreased NK-cell activity (13.74%). Dexamethasone 15 mg/day was initiated.

**Figure 4:**
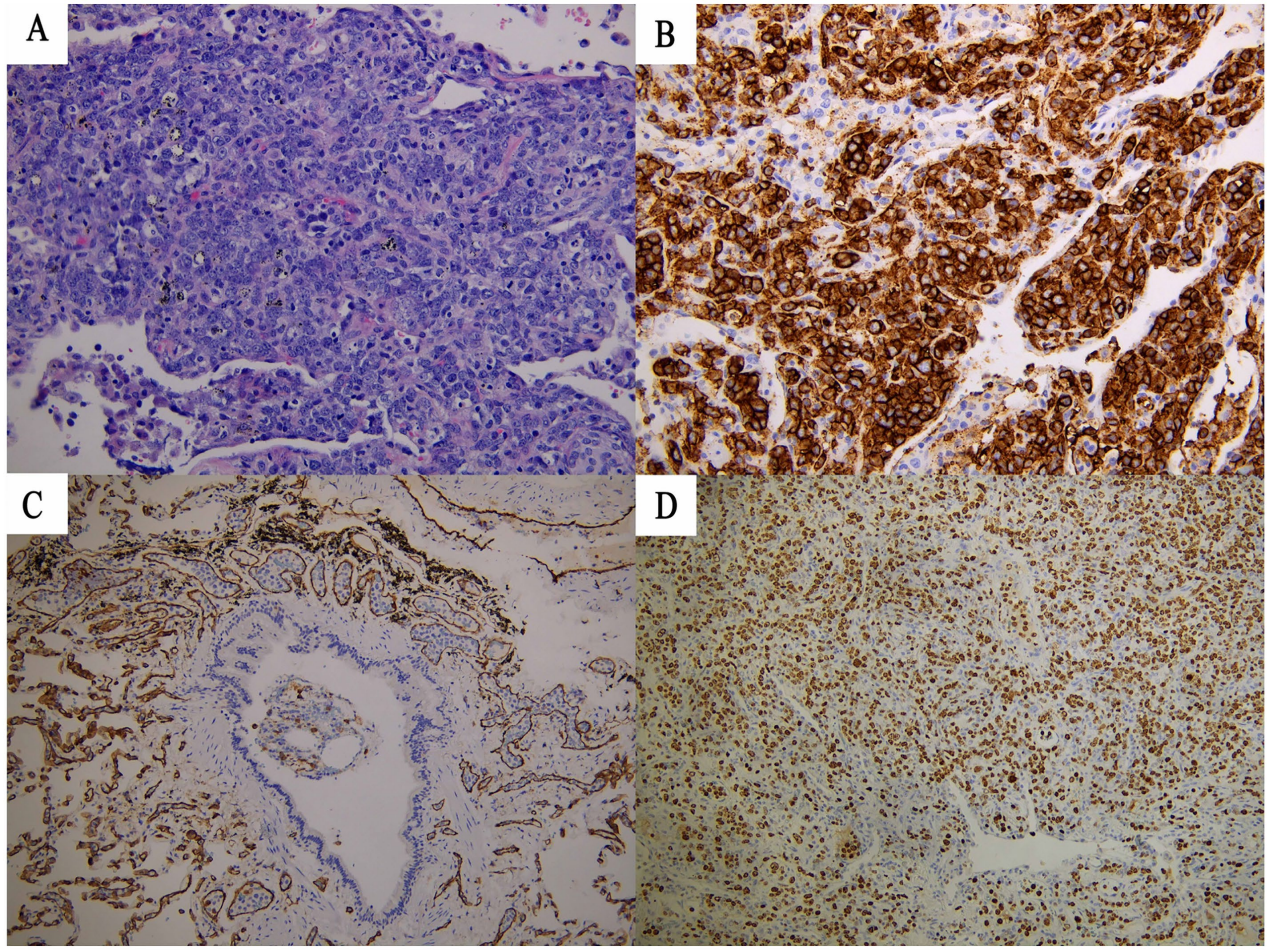
Pathological findings leading to the diagnosis of IVLBCL. **(A)** Morphological assessment (H&E) identifies a lymphomatous infiltrate. Critically, tumor cells are observed both inside vascular spaces and within the interstitium. **(B)** Immunophenotyping confirms a B-cell origin (CD20+). **(C)** The diagnosis of *intravascular* lymphoma is solidified by CD31 staining, which demonstrates tumor cells confined within endothelial-lined vessels. **(D)** Assessment of proliferative activity reveals a markedly elevated Ki-67 index of approximately 70%, consistent with a highly aggressive lymphoma.

Histopathological examination of the lung specimen on August 14 revealed medium to large atypical lymphoid cells within vascular lumina and pulmonary interstitium (Figure 4A). Immunohistochemistry confirmed B-cell lineage (CD20+, PAX-5+; CD3−) (Figure 4B). CD31 staining outlined vascular channels, confirming that the majority of tumor cells were confined within endothelial-lined vessels (Figure 4C). The presence of focal interstitial cells does not exclude IVLBCL, as rare aggressive cases may exhibit extravascular extension due to vascular wall disruption; importantly, the differential diagnosis of reactive intralymphovascular immunoblastic proliferation (ILVIP) was excluded by the strong CD20 and PAX-5 expression and monotypic B-cell phenotype (4). Tumor cells expressed MUM-1, were EBER-negative, and had a high Ki-67 index (~70%) (Figure 4D). The final diagnosis was intravascular large B-cell lymphoma, non-germinal center B-cell phenotype, with secondary hemophagocytic lymphohistiocytosis.

The patient was transferred to Hematology, and on August 20 she commenced R-CHOP immunochemotherapy (rituximab 375 mg/m^2^ day 1, cyclophosphamide 750 mg/m^2^ day 1, doxorubicin 50 mg/m^2^ day 1, vincristine 1.4 mg/m^2^ day 1 [max 2 mg], prednisone 100 mg days 1–5), administered every 3 weeks. Fever resolved within days of starting treatment, and hematologic parameters improved progressively. A follow-up PET-CT on October 24 demonstrated complete metabolic response (CMR) (Figure 3B). At last follow-up on December 31, 2025, the patient remained clinically stable and was continuing planned chemotherapy cycles.

## Discussion

This case exemplifies the profound diagnostic challenge posed by intravascular large B-cell lymphoma (IVLBCL) and offers critical insights for navigating similar clinical dilemmas. IVLBCL represents an important, albeit rare, differential diagnosis for fever of unknown origin (FUO) and secondary hemophagocytic lymphohistiocytosis (HLH) (1, 2).

## The differential diagnosis of FUO: a systematic approach

In patients presenting with prolonged fever, weight loss, and systemic inflammation, the differential diagnosis is broad, encompassing infections, autoimmune diseases, and malignancies (1). Our case illustrates the importance of systematically excluding each category. Infectious causes were rigorously pursued with multiple cultures, NGS of blood and BALF, tuberculosis testing, and fungal markers, all of which remained negative. The lack of response to broad-spectrum antibiotics further reduced the likelihood of bacterial infection. Autoimmune and inflammatory disorders—including systemic lupus erythematosus, vasculitis, and adult-onset Still’s disease—were considered given the presence of fever, arthritis, and elevated inflammatory markers. However, extensive autoantibody testing was unrevealing, and the absence of response to high-dose corticosteroids argued against these diagnoses. Malignancy, particularly lymphoma, emerged as a leading consideration, driven by the constellation of B symptoms, elevated LDH, and hyperferritinemia. Yet, the absence of lymphadenopathy, mass lesions, or definitive findings on random biopsies and PET-CT kept the diagnosis elusive.

## The diagnostic value of the ferritin-LDH-IL-10 triad

The diagnostic pivot in this case arose from interpreting specific laboratory alarms within the clinical context. The triad of extremely elevated ferritin (>5,000 ng/mL), lactate dehydrogenase (LDH), and interleukin-10 (IL-10, rising to >600 pg/mL) served as a powerful beacon. As highlighted by Zhang et al., serum IL-10 serves as a valuable diagnostic and monitoring biomarker for IVLBCL; a cut-off >95.65 pg./mL yields a sensitivity of 80% and specificity of 100% (5). Pronounced IL-10 elevation, linked to an immunosuppressive tumor microenvironment, is strongly suggestive of a lymphoproliferative disorder. Furthermore, extreme elevations of LDH and ferritin are characteristic of IVLBCL (2, 6). Li et al. reported elevated LDH in all patients (20/20, median 780 U/L) and hyperferritinemia in 80% (12/15, median 1,218.8 ng/mL) (6). Similarly, Matsue et al. noted frequent elevations in LDH (median 934 U/L), ferritin (median 1,215 ng/mL), and soluble IL-2 receptor (sCD25) (2).

In our patient, the subsequent development of cytopenias, extreme hyperferritinemia, markedly elevated sCD25 (>44,000 pg./mL), and diminished NK-cell activity unequivocally met the diagnostic criteria for HLH—a known major complication of IVLBCL. Therefore, this “ferritin-LDH-IL-10 triad” in the setting of FUO and systemic inflammation should immediately elevate IVLBCL to the top of the differential diagnosis and prompt urgent evaluation for HLH.

## Limitations of IL-10 as a biomarker

While IL-10 is a promising biomarker for IVLBCL, its elevation is not entirely specific. Elevated IL-10 has been reported in other lymphoma subtypes (including diffuse large B-cell lymphoma and Hodgkin lymphoma), hemophagocytic lymphohistiocytosis (HLH), and severe inflammatory conditions such as systemic lupus erythematosus and active tuberculosis (7). In our patient, the diagnosis of HLH was established concurrently, raising the possibility that IL-10 elevation reflected both tumor-derived production and HLH-associated immune dysregulation. Therefore, IL-10 should be interpreted as part of a broader clinical and laboratory constellation rather than as a standalone diagnostic test.

## Interpreting the triad in the context of HLH: a critical caveat

A critical caveat in interpreting the ferritin-LDH-IL-10 triad is the potential confounding effect of hemophagocytic lymphohistiocytosis (HLH), which frequently complicates IVLBCL. HLH itself drives extreme hyperferritinemia (often >10,000 ng/mL) and marked LDH elevation through macrophage activation and tissue necrosis. Therefore, in patients meeting HLH criteria, the relative contribution of the underlying lymphoma versus the hyperinflammatory syndrome to these laboratory abnormalities cannot be readily disentangled. Nevertheless, IL-10 elevation may help discriminate: while HLH alone can cause moderate IL-6 and ferritin elevations, markedly elevated IL-10 (>600 pg./mL) appears more characteristic of IVLBCL, likely reflecting tumor-derived immunosuppressive cytokine production rather than HLH-driven inflammation alone. This distinction has clinical utility: in a patient with HLH criteria, extreme IL-10 elevation should prompt aggressive pursuit of an underlying lymphoma rather than attribution to HLH alone.

## The limitations of random biopsy and the necessity of image-guided targeting

Confronted with high suspicion but negative routine biopsies, the strategy must evolve from random sampling to deliberate, image-guided targeting. Definitive diagnosis of IVLBCL relies on histopathology, yet its characteristic intravascular growth often leads to low diagnostic yield from conventional random biopsies (e.g., skin, bone marrow) (6). Random skin biopsy (RSB) has established utility, especially in patients with cutaneous lesions or suggestive clinical features (8); however, its yield can be diminished by factors like prior corticosteroid use, as may have occurred in our case.

When clinical suspicion remains high despite negative initial investigations, a meticulous, hypothesis-driven re-interrogation of imaging becomes crucial. In our patient, who presented with cough and impaired gas exchange yet an unremarkable chest CT—a presentation reminiscent of pulmonary IVLBCL cases reported even without definite PET-CT abnormality (9)—the pivotal step was the identification of a discrete, albeit metabolically subtle (SUVmax 3.0), pulmonary focus upon re-review of PET-CT. This finding mandated biopsy, fully aligning with the principle that when imaging reveals a suspicious lesion, biopsy should be directed at that site (8). It underscores that any identifiable lesion, regardless of its metabolic intensity, becomes the prime target when pursuing this intravascular “great imitator” (7). For patients presenting with abnormal high-resolution computed tomography (HRCT) findings, transbronchial lung biopsy (TBLB) may also serve as a valuable diagnostic tool (10).

## Interpreting low-grade FDG avidity (SUVmax 3.0)

The FDG-avid pulmonary focus in our patient had a maximum standardized uptake value (SUVmax) of 3.0, a level that typically falls within the range observed in inflammatory conditions such as infection, sarcoidosis, or organizing pneumonia. Several factors supported pursuing biopsy despite the low SUVmax. First, the clinical context—persistent FUO, extreme hyperferritinemia, markedly elevated IL-10, and failure of empirical antimicrobial and anti-inflammatory therapies—strongly favored an underlying neoplastic process over infection or autoimmunity. Second, the pattern of FDG uptake (diffuse bilateral pulmonary involvement with subtle focal accentuation) was atypical for common infections, which typically show more intense and localized uptake. Third, re-review by a multidisciplinary team identified this focus as the only site of metabolic abnormality after excluding physiological variants. These considerations illustrate that in the appropriate clinical context, even low-grade FDG avidity can be a valid diagnostic target.

## Why earlier biopsies were negative and why targeted lung biopsy was pursued

The diagnostic yield of random biopsies in IVLBCL is inherently limited by the patchy, intravascular nature of the disease. Reported diagnostic yields vary: random skin biopsy (RSB) has a sensitivity of approximately 50–80% in patients with cutaneous manifestations, but this declines to <30% in those without skin lesions, particularly when performed after corticosteroid exposure (8). Bone marrow biopsy, while commonly performed in FUO workups, is diagnostic in only 30–40% of IVLBCL cases, as tumor cells often spare the marrow space or infiltrate minimally (2). Random gastrointestinal biopsies have an even lower yield (<10%) in the absence of macroscopic abnormalities, as illustrated by our patient’s four negative colonoscopies (6).

The decision to pursue VATS lung biopsy was driven by three converging factors: (1) persistent unexplained hypoxemia and respiratory symptoms despite normal chest CT, raising suspicion for microvascular pathology; (2) re-review of PET-CT revealing a subtle but definite FDG-avid focus (SUVmax 3.0) in the right upper lobe; and (3) failure of all less-invasive diagnostic modalities. Even low-grade FDG avidity (SUVmax 3.0) can be diagnostic in the appropriate clinical context. PET-CT has emerged as a powerful tool for guiding biopsy in IVLBCL: a recent study of 42 IVLBCL patients found that 73.8% showed FDG-avid lesions, with a median SUVmax of 7.4 (range 1–27.7) (11). Notably, some patients with IVLBCL have negative PET-CT findings, in which case random skin biopsy remains essential.

## Practical lessons for clinicians

From this case, clinicians should take away three actionable lessons: (1) In FUO with extreme hyperferritinemia and elevated LDH, suspect HLH and investigate for underlying lymphoma, with IL-10 as a potentially discriminatory biomarker. (2) When random biopsies are negative but suspicion remains, re-review imaging carefully—even subtle PET-CT abnormalities (SUVmax as low as 3.0) can be diagnostic targets. (3) Multidisciplinary team evaluation is not merely academic; it directly enabled the diagnostic biopsy in this case by integrating pulmonary, nuclear medicine, and surgical expertise.

## The indispensable role of multidisciplinary collaboration

In such complex diagnostic scenarios, the role of a multidisciplinary team (MDT) is indispensable. Faced with persistent fever, unexplained respiratory symptoms, and inconclusive results from multiple investigations—including bronchoscopy, bone marrow aspiration, and initial PET-CT—an MDT comprising radiology, respiratory, thoracic surgery, hematology, and pathology was convened. Despite the absence of distinct structural abnormalities on chest CT, the team integrated the clinical, laboratory, and imaging data, with particular attention to the subtle but suggestive metabolic focus on PET-CT. This collaborative evaluation led to the decisive recommendation for a targeted lung biopsy. This case highlights that MDT-driven, hypothesis-guided targeting of even subtle imaging abnormalities is essential for diagnosing elusive entities like IVLBCL, especially when conventional approaches have failed.

## Clinical heterogeneity of IVLBCL

IVLBCL commonly manifests as FUO, multisystem involvement, and marked laboratory abnormalities (e.g., elevated LDH, ferritin, and sCD25). A 20-year single-center Japanese study of 42 patients documented a high prevalence of persistent fever (97.6%), hypoalbuminemia (100%), hypoxemia (80.0%), thrombocytopenia (81.0%), and anemia (69.0%) (2). The so-called “Western variant” is described as more frequently involving the central nervous system and bone marrow (3) while the “Asian variant” is associated with hematologic abnormalities and hemophagocytic syndrome (2).

Notably, our patient’s presentation—dominant pulmonary symptoms, FUO, fulminant HLH, and occult hypoxemia—diverges from these classic patterns, aligning more closely with the rare “primary pulmonary IVLBCL” subtype reported in three cases from Nanjing Drum Tower Hospital, which presented with fever, cough, and progressive dyspnea (12). This heterogeneity explains the initial failure of random biopsies (bone marrow, skin, gastrointestinal) and underscores that atypical presentations are the rule rather than the exception in IVLBCL.

## Conclusion

This case illustrates that while individual elements of our approach—IL-10 as a biomarker, PET-CT guidance for biopsy, and multidisciplinary collaboration—have been described separately, the novel contribution lies in their integration into a coherent diagnostic algorithm for navigating the diagnostic impasse when random biopsies fail. The triad of extreme hyperferritinemia, elevated LDH, and markedly increased IL-10 should be interpreted as a potential diagnostic clue that raises suspicion for IVLBCL in FUO patients, bearing in mind the confounding effects of secondary HLH. When routine investigations and random biopsies are unrevealing, a targeted, imaging-guided approach—centered on careful PET-CT review and strategic biopsy of metabolically active sites—proved essential for definitive diagnosis. Early multidisciplinary collaboration among infectious diseases, hematology, radiology, and pathology is crucial to accelerate diagnostic clarity and initiate timely, life-saving therapy.

## Data Availability

The original contributions presented in the study are included in the article/supplementary material, further inquiries can be directed to the corresponding author/s.
